# Need, demand, and feasibility for a new iNTS vaccine: stakeholder insights from eight African countries

**DOI:** 10.1038/s41541-026-01391-2

**Published:** 2026-02-11

**Authors:** Sajan Gunarathna, Yongha Hwang, Jung-Seok Lee, GeunHyeog Jang, Jean-Louis Excler, Florian Marks, Jerome H. Kim

**Affiliations:** 1 International Vaccine Institute, Seoul, Republic of Korea; 2 College of Natural Sciences, Seoul National University, Seoul, Republic of Korea

**Keywords:** Diseases, Health care, Microbiology

## Abstract

This study, grounded in the key stakeholders’ perspectives, highlights the critical and immediate need for the development of a new iNTS vaccine. An online survey was conducted among government officials and healthcare providers across eight African countries: Burkina Faso, the Democratic Republic of Congo, Ethiopia, Ghana, Kenya, Malawi, Mozambique, and Nigeria. The framework was built on Spring Boot and Java 8, enabling access via personal computers or mobile phones. A total of 74 out of 84 participants completed the survey. Over 50% indicated that iNTS is both a prevalent and serious disease in their respective countries. The majority (*n* = 70, 94.6%) identified antibiotics as the most effective treatment for iNTS disease, while 55 (67.6%) participants chose vaccination as the preferred preventive option. Respondents ranked the iNTS vaccine third. Notably, 75.7% (*n* = 56) emphasized the urgent need for an iNTS vaccine. Barriers to vaccine introduction were identified as insufficient funding (*n* = 61, 81.4%), limited awareness (*n* = 49, 79.7%), and challenges related to community acceptance (*n* = 49, 79.7%). The stakeholders’ perspectives highlight an urgent need for iNTS vaccine development. Accelerated and focused efforts are essential to address the reported pressing challenges and to pursue innovative and promising pathways for advancing iNTS vaccine development.

## Introduction

Invasive non-typhoidal *Salmonella* (iNTS) disease is a bacterial infection caused by the *Salmonella* Genus. Non-typhoidal S*almonella* (NTS), that most commonly presents as diarrhoeal illness, can invade sterile sites in the human body, resulting in iNTS with various features including bacteraemia, meningitis, organ abscesses, septic shock and other focal infections^1,2^. Therefore, iNTS is categorized as a bacterial infectious disease caused by extraintestinal infection of non-typhoidal serotypes of *Salmonella enterica*^1,3,4^. Patients typically present symptoms of fever, pallor and respiratory signs such as cough, tachypnea, and pneumonia^1,5^. The most vulnerable population is children with malnutrition, malaria, sickle-cell anemia and human immunodeficiency virus (HIV) infection, as well as immunocompromised adults with HIV^4^.

The iNTS disease is considered one of the primary causes of global morbidity and mortality. By age group, the disease burden is high among infants and young children (<10 years), and elderly iNTS patients (<50 years) with septicemia, pneumonia, or osteomyelitis also resulted in a higher length of hospital stay and mortality rate^6^. According to the Institute for Health Metrics and Evaluation (IHME) estimation, the global disease burden from iNTS in 2021 was 510,000 cases, 62,000 deaths and 4.74 million global disability-adjusted life years^7^. The total disease burden of iNTS is still under-reported in the African region. However, existing evidence reports an annual incidence of 175–388 cases per 100,000 children aged 3–5 years^8–10^ and 2000–7500 cases per 100,000 HIV-infected adults^11–14^. In sub-Saharan Africa, iNTS infections are a more significant cause of bacteremia compared to other parts of the world^4^.

Many reports have indicated the associated reasons behind this trend. First, HIV/AIDS and malaria, both highly prevalent in these regions, cause immune suppression that increases susceptibility to iNTS. Second, poor sanitation and hygiene, coupled with limited access to clean water and proper sanitation facilities, increase the risk of bacterial infections. Third, malnutrition, particularly among children, compromises immune systems, making them more vulnerable to infections. Fourth, delays in diagnosis and treatment due to limited access to healthcare services exacerbate the situation^15^. Finally, multidrug-resistant strains of iNTS complicate treatment and control efforts. This resistance makes it challenging to manage the disease effectively, further exacerbating the health crisis in the African region^16^.

Given the high disease and economic burden of iNTS disease, controlling the disease should be a top priority, especially in resource-limited settings, such as in the African region^7^. Antibiotic therapy is the current primary treatment, but finding an alternative method is crucial as increased antibiotic consumption leads to antimicrobial resistance (AMR)^17^. Vaccination is a promising preventive alternative; however, to date, no licensed iNTS vaccines are available for human use^18^. The development of effective vaccines is necessary and holds the potential to significantly reduce case numbers and economic burden and minimize antibiotic use^19,20^.

While the absence of a vaccine against iNTS disease is a critical issue, the crucial question remains: is there an immediate need for a new vaccine? This query is of utmost importance due to the scarcity of health care resources and the inherent opportunity cost considering other competing health problems. Therefore, gathering insights from policymakers/stakeholders in high-burden countries is imperative to fuel the vaccine development processes for both private and public sectors. It is worth noting that such evaluations are scarce in the available literature. Hence, this study aims to gauge the perceptions of key stakeholders, focusing on their views on the severity of the disease and the urgency and importance of developing a new vaccine.

## Results

### Socio-demographic information of the respondents

A total of 84 participants initially accessed the online survey, of whom 74 completed it. Only those who completed the survey were included in the analysis. The mean age was 40 years, and 55.4% (*n* = 41) were males. Over 60% were medical doctors or researchers. Participants had varying years of experience, with most holding graduate-level degrees (Table 1).

**Table 1:** Socio-demographic information of the respondents

Characteristic	Statistics	
Age	Mean (Standard deviation)	40 (9.8)
Gender [*n* (%)]	Male	41 (55.4)
Number of participants per country [*n* (%)]	Burkina Faso	9 (12.2)
	Democratic Republic of Congo (DRC)	25 (33.8)
	Ethiopia	4 (5.4)
	Ghana	10 (13.5)
	Kenya	3 (4.1)
	Malawi	11 (14.9)
	Mozambique	1 (1.4)
	Nigeria	11 (14.9)
Types of institute/organization [*n* (%)]	Ministry of Health	19 (25.7)
	Public health facility	24 (32.4)
	Private health facility	11 (14.9)
	Other governmental offices	20 (27.0)
Occupation [*n* (%)]	Medical doctor/ clinician	29 (39.2)
	Researcher	26 (35.1)
	Nurse	6 (8.1)
	Governmental official	1 (1.4)
	Others^a^	12 (16.2)
Years of experience in the current field [*n* (%)]	>15 years	18 (24.3)
	11–15 years	11 (14.9)
	6–10 years	21 (28.4)
	1–5 years	24 (32.4)
Level of education [*n* (%)]	Graduate school -Doctor of Medicine (MD)/Doctor of Philosophy (PhD)	29 (39.2)
	Graduate school – master’s degree	31 (41.9)
	Undergraduate	13 (17.6)
	10–12 years of school	1 (1.4)

^a^Health advisor, medical laboratory scientist, physician biologist, public health pharmacist, public health program manager, research laboratory scientist, biomedical technologist

### Perception and knowledge of iNTS disease

A total of 95.9% (*n* = 71) had heard of iNTS disease. Among the primary symptoms of iNTS (fever and cough), majority identified fever as a correct symptom (*n* = 73, 19.1%), while least participants identified cough as a primary symptom (*n* = 11, 2.9%). Among the given list of incorrect symptoms of iNTS (sore throat, exhaustion, headache, rash, diarrhoea, myalgia, vomiting, and anorexia), most participants identified diarrhoea (*n* = 63, 16.5%) and vomiting (*n* = 63, 16.5%) as correct symptoms (Supplementary Table 1).

Moreover, while a substantial number of participants correctly identified direct (host) risk factors, such as malaria, HIV, malnutrition, and sickle cell anemia, a greater proportion selected indirect (environmental) risk factors including unsafe drinking water (*n* = 63, 16.6%), consumption of contaminated foods (n = 62, 16.4%), and inadequate hygiene and sanitation practices (*n* = 62, 16.4%). Notably, some respondents also cite incorrect risk factors, such as flies (*n* = 36, 9.5%), and direct person-to-person transmission (*n* = 24, 6.3%) (Supplementary Table 1).

Among the 10 practices given to prevent the iNTS disease, the majority (*n* = 71, 17.5%) identified maintaining proper personnel hygiene by washing hands frequently, while 50 participants (12.3%) identified vaccination as the best prevention method. Vaccination ranked sixth among the listed practices. Most respondents (*n* = 70, 94.6%) identified antibiotics as the most effective treatment, whereas 21 participants (12.1%) wrongly classified vaccination as a treatment option. Notably, vaccination ranked fourth among the choices given, following antibiotics, cleaning the house/environment/body and drinking an adequate amount of clean water (Supplementary Table 1).

Furthermore, infants and children up to 15 years of age were most frequently identified as the highest-risk group for iNTS disease (Supplementary Table 1).

**Fig. 1:**
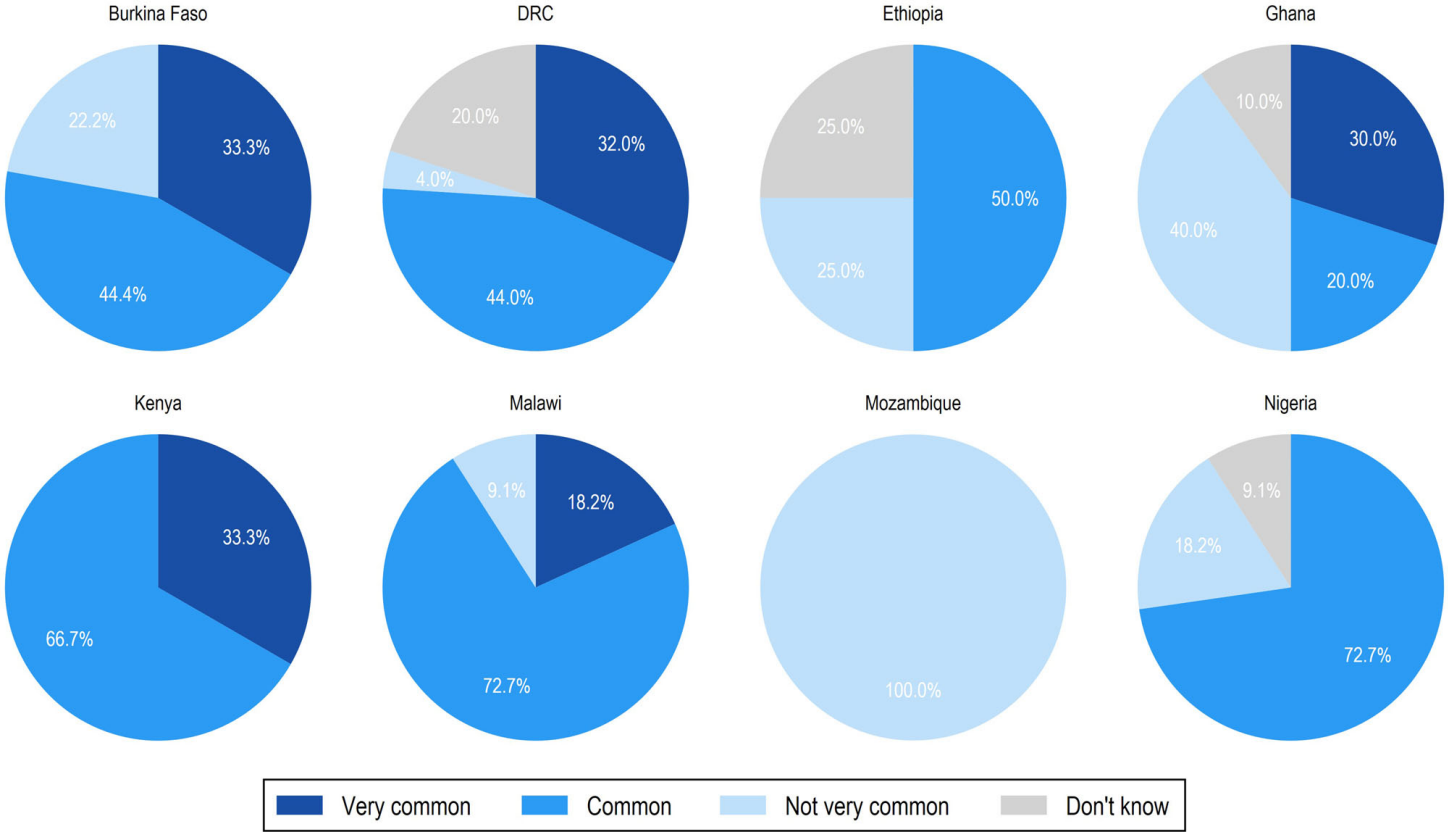
Stakeholder perceptions of the commonness of iNTS disease across countries. Country-specific pie charts show the perceived frequency of iNTS disease in the selected 8 African countries, classified as very common, common, not very common, or don’t know. Values represent the percentage of respondents per country.

Overall, 50% and 23% of respondents indicated that iNTS disease is common or very common in their country, respectively. At the country level, ~30% of respondents from Burkina Faso, DRC, Ghana, and Kenya reported iNTS disease as being very common, while ~20% of respondents reported it as very common in Malawi. Notably, in Mozambique, the disease was perceived as less prevalent relative to the other countries; however, this observation is based on a single respondent (Fig. 1).

**Fig. 2:**
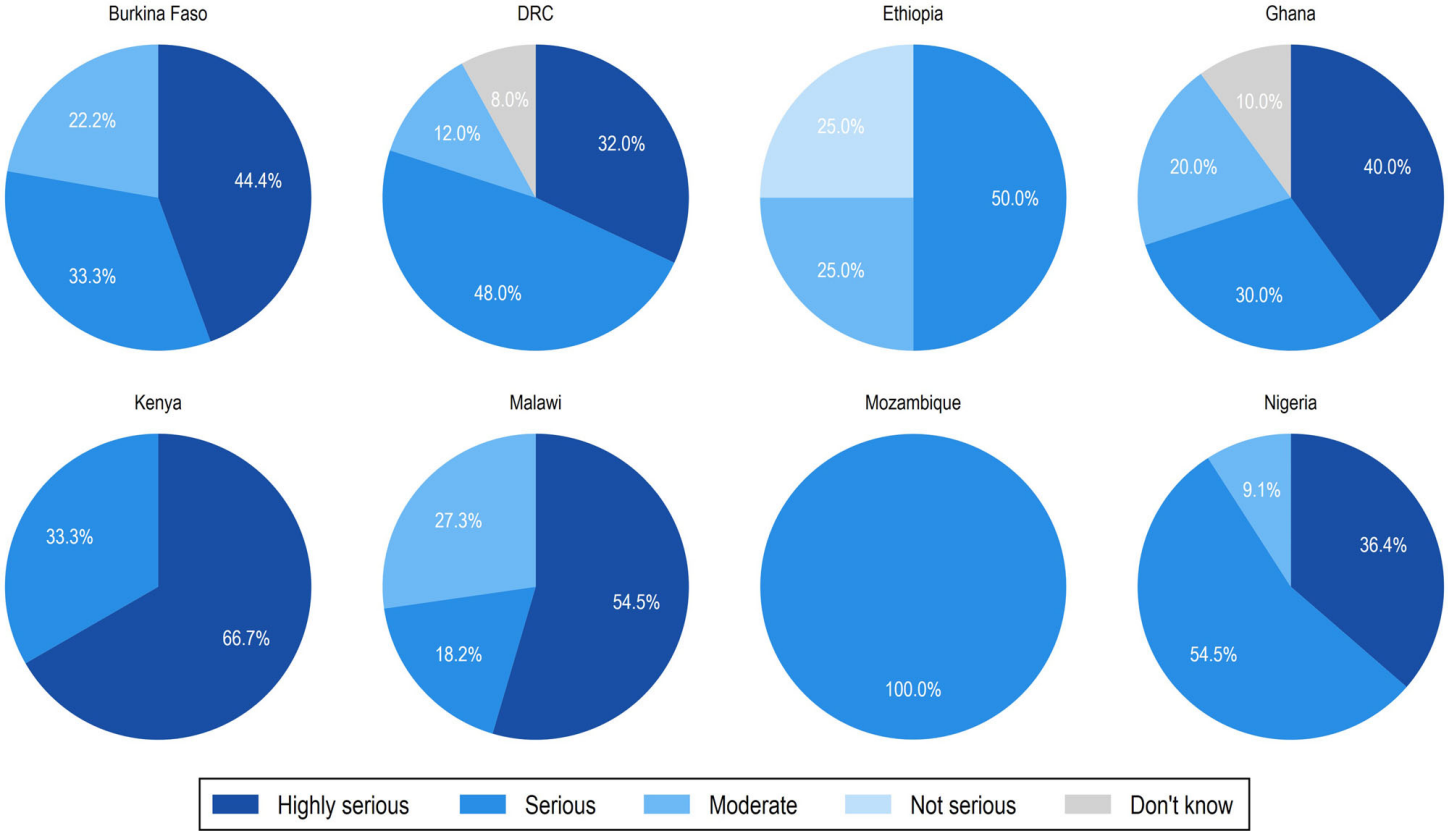
Stakeholder perceptions of the seriousness of iNTS disease across countries. Country-specific pie charts show respondents’ assessments of iNTS disease severity in the selected 8 African countries, categorized as highly serious, serious, moderate, not serious, or don’t know. Values represent the percentage of respondents per country.

Among all respondents, 37.8% and 40.5% perceived iNTS disease as very serious or serious, respectively. Participants from Kenya (66.7%), Malawi (54.5%), Burkina Faso (44.4%), Ghana (40.0%), Nigeria (36.4%), and DRC (32.0%) reported iNTS disease as very serious in their respective countries (Fig. 2).

### Vaccines and iNTS disease

**Fig. 3:**
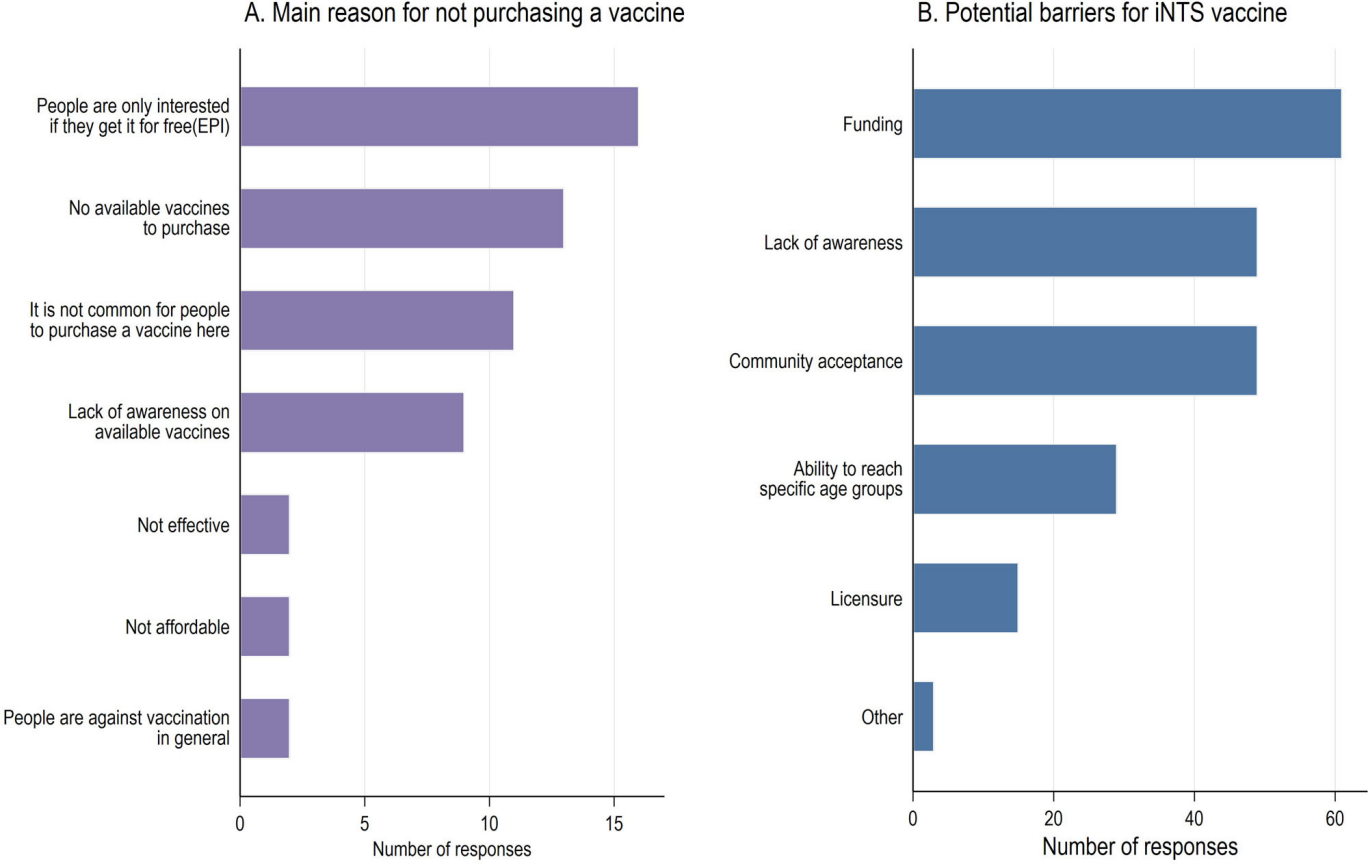
Perceived reasons for not purchasing vaccines and barriers to the introduction of an iNTS vaccine. **A** Main reasons reported by respondents for not purchasing vaccines. **B** Key perceived barriers to iNTS vaccine introduction. Bars indicate the number of responses for each reason or barrier.

A total of 74.3% (*n* = 55) of respondents claimed that people never purchase any vaccines in their area. All respondents from Ethiopia and Mozambique reported that vaccines had never been purchased in their communities. However, respondents from other countries reported instances of vaccine purchases, including Kenya (66.7%), Nigeria (45.5%), Burkina Faso (44.4%), Ghana (30%), Malawi (27.3%), and DRC (8%). The most frequently cited reasons for not purchasing vaccines included: ‘interest only in free vaccinations’, ‘no available vaccines to purchase’, and ‘uncommon to purchase vaccines in the country’. Moreover, funding, lack of awareness, and community acceptance were the most indicated barriers to iNTS vaccine introduction (Fig. 3).

A total of 64.9% of respondents identified routine and catch-up vaccination as the most appropriate vaccination strategy. More than half of the participants (52.1%) responded that achieving a coverage rate of 90% or higher for both routine and catch-up vaccinating programs is necessary for effective prevention of iNTS disease (Supplementary Fig. 1).

Regarding government involvement, most of the respondents (95.9%) believed that, if an iNTS vaccine becomes available, their governments would consider it to be a part of EPI. This perception was primarily driven by three elements: “vaccines are useful for the general public because they are good for prevention and safety” (58.1%), “iNTS is a dangerous disease” (20.3%), and “the burden of iNTS disease is high” (17.6%). As for the preferred vaccination schedule, a two-dose scheme emerged as the most favored option; however, the differences in preference among the single-dose, two-dose, and three-dose schemes were minimal.

**Fig. 4:**
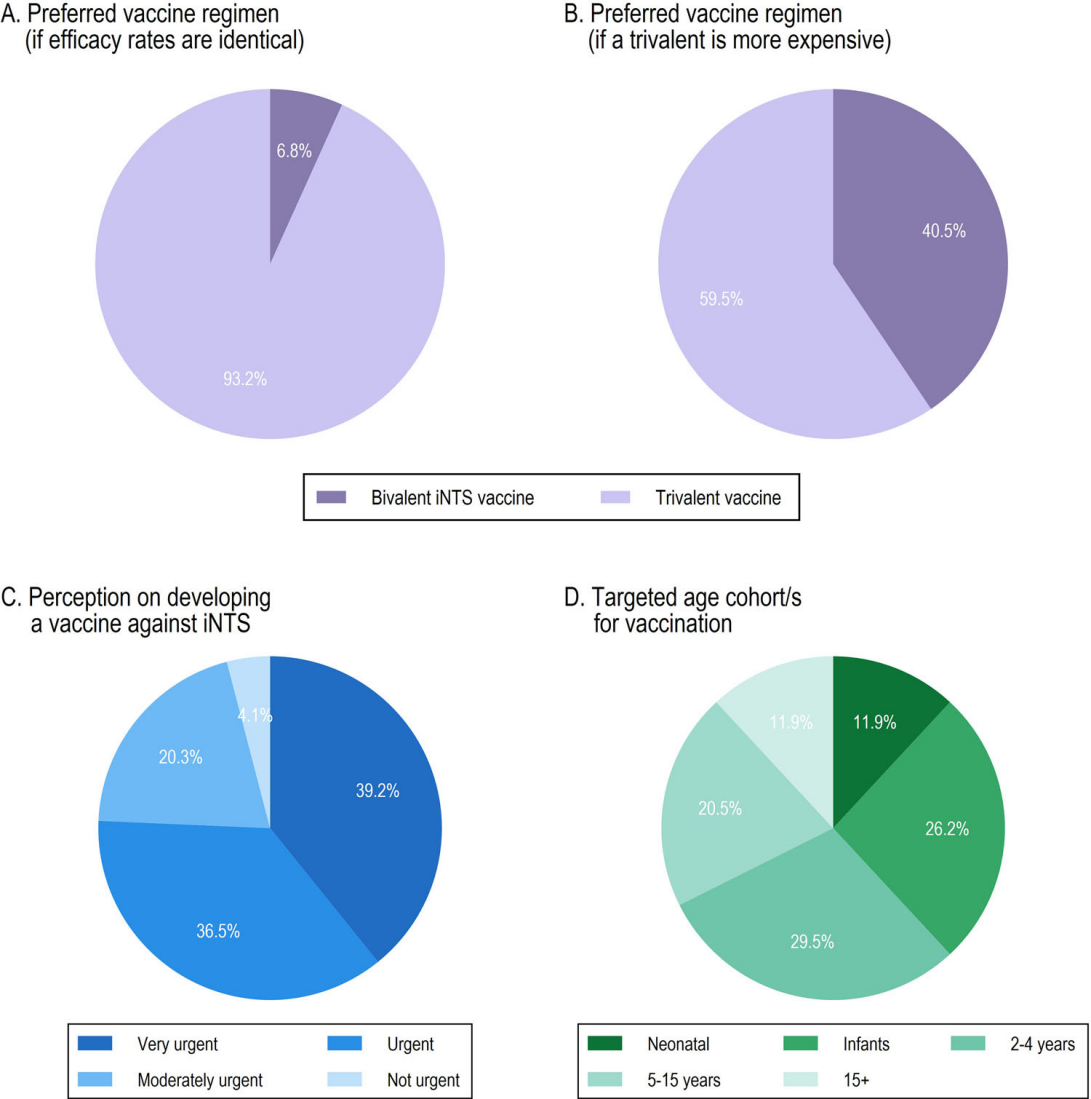
Stakeholder preferences and perceptions related to iNTS vaccine introduction. **A** Preferred vaccine regimen assuming identical efficacy between bivalent and trivalent iNTS vaccines. **B** Preferred vaccine regimen if the trivalent vaccine is more expensive. **C** Perceived urgency of developing an iNTS vaccine. **D** Preferred target age cohorts for iNTS vaccination. Pie charts show the percentage of respondents selecting each option.

Over 90% of the respondents reported a preference for a 3-dose vaccine over a 2-dose one, assuming equal efficacy between the two. However, this preference shifted significantly when cost differences were introduced; the proportion favoring the 2-dose vaccine rose from 6.8% to 40.5% if the 3-dose vaccine were more expensive (Supplementary Table 2). In terms of target age groups for vaccination, younger cohort (<5 years) were prioritized. Within this group, vaccinating neonates was less popular and ranked third after the cohorts of 2–4 years of age and infants (Fig. 4).

### Importance of iNTS vaccine

**Fig. 5:**
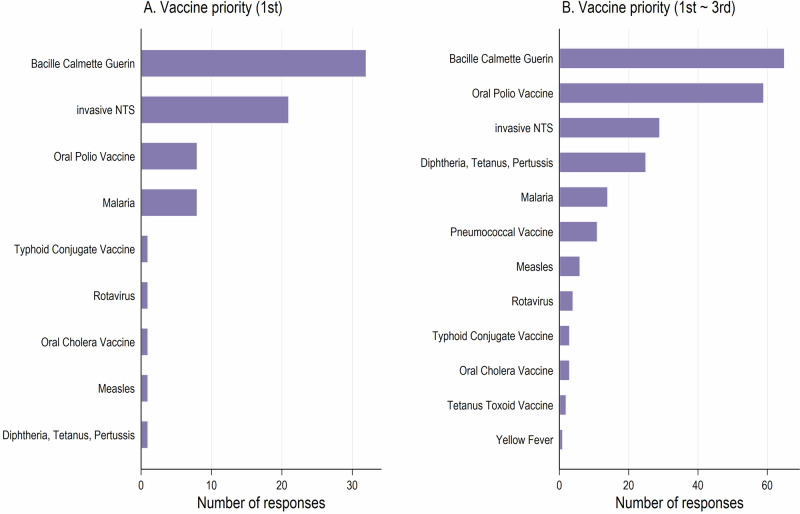
Vaccine priority. **A** Vaccines ranked as the highest priority (considering only the first choice) by respondents. **B** Vaccines ranked within the top three priorities by respondents. Bars indicate the number of responses for each pre-specified list of vaccines provided by the study team.

Respondents were asked to rank the importance of an iNTS vaccine relative to other existing vaccines. A total of 21 respondents identified the iNTS vaccine as their highest priority, ranking it second only to the Bacille Calmette Guerin (BCG) vaccine, which was prioritizes by 32 respondents. When considering their top three priorities, 29 respondents placed the iNTS vaccine in third position. In comparison, 65 respondents prioritized the BCG vaccine, followed by 59 prioritizing the oral polio vaccine (Fig. 5). Furthermore, 75.7% (*n* = 56) of respondents reported the development of iNTS vaccine to be urgent, with 39.2% (*n* = 29) rating it as very urgent.

## Discussion

The present study captures the perspectives of 74 key stakeholders within the health care sector in selected countries from the African region, concentrating on their assessments of the gravity of iNTS disease and the pressing need for a novel vaccine. Awareness of iNTS was high. However, the critical issue lies in the accuracy of their understanding of the associated symptoms of iNTS disease as in the absence of strong evidence to support these perceptions.

Approximately one-third of the respondents asserted that the iNTS disease is more widespread in their respective countries and also perceived the disease as not only more common but also more severe in these nations. The higher incidence of the disease in these countries could be attributed to several common health-related factors in the African region, such as high prevalence of HIV/AIDS and malaria, poor sanitation and hygiene, malnutrition, delays in diagnosis and treatment^15^, and the prevalence of the multidrug-resistant strains of iNTS^16^. The global and African regional data from 2005 to 2023 suggest that iNTS disease decreased over time^21^. However, the case fatality ratio remained high, and multidrug-resistant strains were frequently detected, complicating treatment efforts^22^.

Most respondents identified antibiotics as the optimal treatment for iNTS disease. While they regard this as a crucial option, the excessive use of antimicrobial drugs contributes to AMR, resulting in significant health and economic challenges. The World Health Organization (WHO) has projected that antibiotic overuse could lead to 10 million deaths by 2050 and push 24 million people into extreme poverty by 2030^23^. Given the substantial disease and economic burden of AMR, prioritizing preventive is imperative. Most respondents identified in-house preventive measures, such as maintaining proper personal hygiene by frequently washing hands and boiling water before use, as the best preventive strategies. While these practices are crucial for preventing many infections, they may not be sufficient on their own to prevent iNTS disease. Comprehensive preventive measures, including vaccination, are necessary for effective prevention^24^. Nearly two-thirds of participants considered iNTS vaccine as the best preventive measure, emphasizing the importance of high coverage rates for routine and catch-up vaccines. This underscores a clear priority towards preventive measures, as vaccines are instrumental in controlling and eliminating life-threatening infectious diseases, providing long-term protection^4^. It is worth noting that the price of a vaccine plays an important role in the context of the 2-dose versus a 3-dose vaccination regimen. A 3-dose vaccination regimen was generally preferred over a 2-dose regimen with half of the respondents supporting its universal introduction. However, the higher price for this schedule makes a 2-dose regimen more attractive in many countries.

In the present study, respondents ranked the importance of the iNTS vaccine as the third highest, following the BCG vaccine and OPV. This prioritization likely stems from the high incidence rates of tuberculosis, which is one of the significant health concerns in the African region^21^. Despite the region’s major achievement of being certified free of indigenous wild polio in 2020^25^, polio vaccination remains a top priority. However, the vaccine priority results should be interpreted with caution, as they differ from the WHO priority list^26^, and are inconsistent with the priorities of the African Vaccine Manufacturing Accelerator (AVMA)^27^.

When evaluating vaccines as a valuable option, it is essential to identify the countries with the highest burden, as interventions may fail due to the lack of understanding of specific country or regional priorities. According to our findings, people in the region are unlikely to purchase vaccines due to a preference for free vaccinations, the unavailability of vaccines for purchase, and a lack of awareness about available vaccines. Funding issues and community acceptance were also identified as major barriers to the implementation of such interventions. Beyond these challenges, the region faces supply issues, inadequate infrastructure, logistical difficulties, and poor production capacity, as also highlighted by WHO^28,29^. However, most respondents indicated that the government is a significant strength, as it would consider iNTS vaccine to be a part of the EPI due to its understanding of the severity of iNTS disease and the importance of prevention and safety.

Our study findings may offer the public health authorities, NITAGs and EPI programmes ways to undertake a structured assessment of the key barriers highlighted in this study—such as limited funding, lack of awareness, and concerns about community acceptance—and map context-specific pathways to address them. Moreover, policymakers may consider investigating priority populations and delivery platforms for a future iNTS vaccine and budget-impact and operational analyzes that reflect stakeholders’ concerns about price and feasibility. Country-specific evidence generated from such investigations will help plan incremental investments in logistics and human resources to accommodate an additional vaccine; and design targeted communication and community engagement strategies.

The study has several strengths. It was guided by a series of questions that are poorly addressed in literature. We used a systematic, detailed, and transparent study method, enhancing the reliability and validity of the study findings by minimizing biases in data collection, analysis, and interpretation. First, we employed a standardized questionnaire featuring clear, simple, and unambiguous wording to ensure consistency in administration and to avoid leading or double-barreled questions. Second, the questionnaire was reviewed by several senior economists, and revisions were made based on their expert feedback. Third, we conducted a pilot test and further refined the questionnaire to address any issues related to ambiguity, relevance, or wording.

However, the findings from this study need to be understood with the following limitations. First, given that the current study employed a key-informant survey based on purposive sampling, the study findings are limited to the eight countries in the African region with descriptive statistics. Second, while a total of 74 respondents completed the survey, Mozambique with only one participant, was marginally presented and the sample size from DRC was disproportionately higher compared to the others. Third, the findings should be regarded as indicative of expert opinion rather than fully generalizable to all contexts and stakeholder groups. Finally, the respondents’ observative and adaptive behavior could not be eliminated according to the survey type, as they were given total freedom to complete the survey online remotely, where they could reference checking and consulting secondary sources.

At the conclusion, the current study highlighted the gaps in the awareness and understanding of iNTS disease. While most respondents recognized the disease, some were misinformed about its symptoms, some of which are not currently listed as associated symptoms. Overall, iNTS disease was seen as particularly prevalent and severe in Burkina Faso, DRC, Ghana, Kenya, Malawi and Nigeria. Antibiotics were widely regarded as the best treatment, while maintaining personal hygiene was considered crucial for prevention. Vaccination emerged as a critical preventive measure, though challenges such as funding, community acceptance, and logistical issues were significant hurdles in this region.

The iNTS vaccine was considered the third most critical vaccine, after the globally essential BCG and OPV vaccines. This underscores the severe impact of iNTS disease, highlighting its urgency and the pressing need for a safe and effective vaccine to prevent this disease. Hence, minimizing the identified challenges the countries face needs to be considered, and searching possible pathways to accelerate the development of a new iNTS vaccine should be a priority.

## Methods

This study adhered to the Strengthening the Reporting of Observational Studies in Epidemiology (STROBE) guidelines for cross-sectional studies to ensure transparency in reporting^30^ (Supplementary Table 3).

### Survey design

An online survey was designed to inform policymakers of the need, demand for, and feasibility of a vaccine against iNTS disease. To understand general perceptions on iNTS infections and future vaccines against the disease, the survey consisted of three sections in the following domains: (1) background information for participants, (2) perceptions and knowledge of iNTS disease, and (3) vaccine development. The survey was conducted from May to June 2024. Considering the multi-country contexts, the survey was conducted in English, French, as well as in Portuguese.

### Survey system and implementation

To help respondents participate in the survey with easy access to the online system, Spring Boot and Java 8 were utilized to construct the framework of the survey tool; and JavaScript, Hyper Text Markup Language 5 (HTML 5), Cascading Style Sheets and Java Persistence API (JPA) were used for the back- and front ends. The respondents were able to participate in the survey via their personnel computers (PCs) or mobile phones with the ability to start, stop, and resume the survey any time during the survey period with a temporary save feature.

**Fig. 6:**
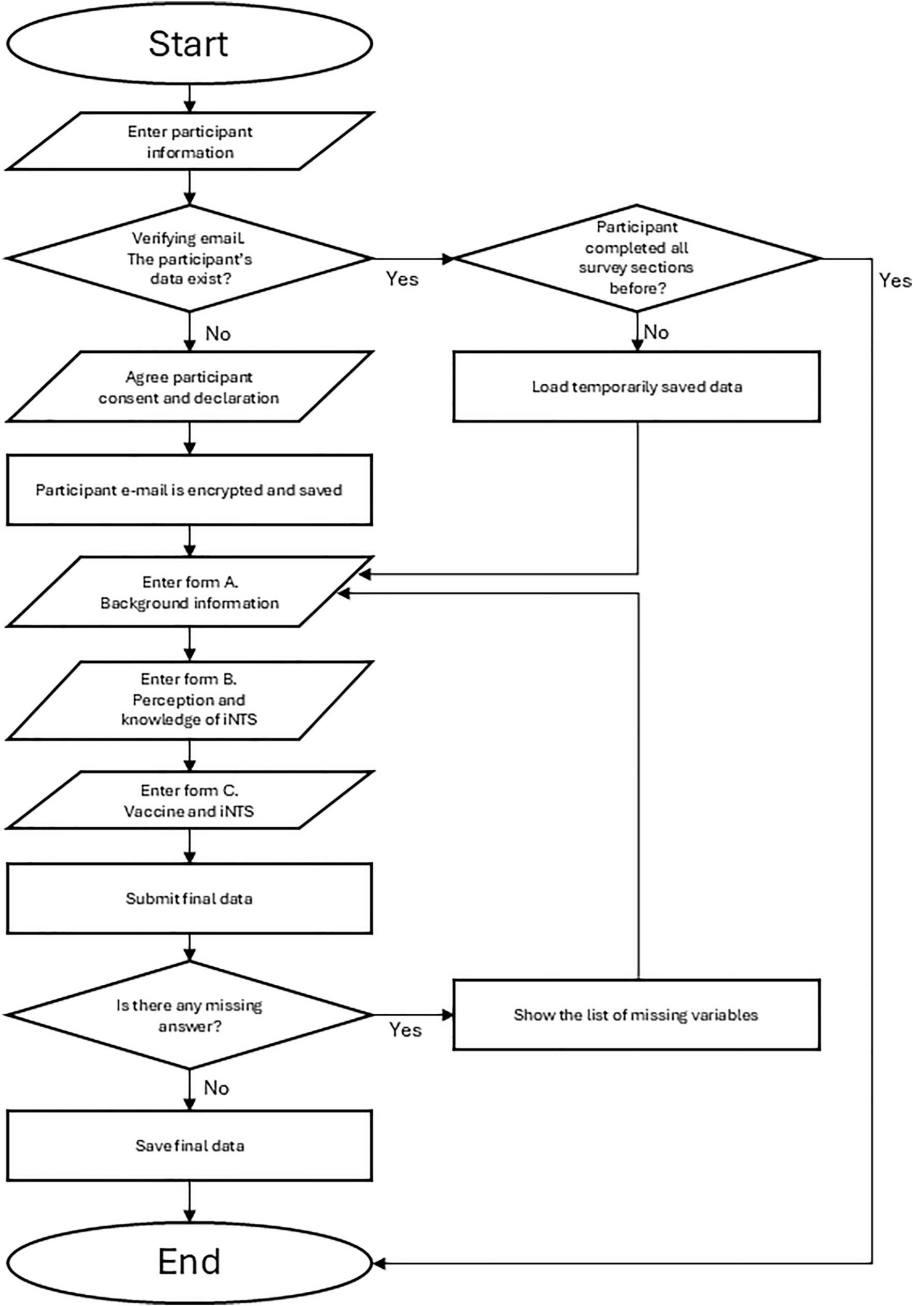
Survey flowchart. The diagram illustrates participant enrolment, consent, data verification, survey completion across three sections (background information; perceptions and knowledge of iNTS; and vaccines and iNTS), validation of missing responses, and final data submission and storage.

The overall structure of the current survey system is shown in Fig. 6. On the main (first) page of the survey tool, a participant was asked to enter general information, such as email, country, and date of survey. All fields were mandatory to verify a double-entry, and the survey identification (ID) number was automatically generated based on the general information. The email address was encrypted by the Secure Hash Algorithm 256 before the data was saved in the database, thus no one could track the participant’s original email address.

When the participants submitted their responses with any missing values, the system showed a pop-up message indicating the variables with missing values, and the participants were asked to go back to the questions and answer them. Once the participant submitted the final responses with no missing value, the survey was considered completed.

### Survey participants: inclusion and exclusion criteria

Public health authorities, health care providers, including country Essential (formerly Expanded) Programme on Immunization (EPI) leads; new vaccine officers; and National Immunization Technical Advisory Group (NITAG) members in eight African countries (Burkina Faso, Democratic Republic of Congo (DRC), Ethiopia, Ghana, Kenya, Malawi, Mozambique, and Nigeria) were eligible for inclusion. Individuals who voluntarily consented to participate were included in the study.

Respondents who did not complete the survey or who could not read or speak any of the three survey languages (English, French, or Portuguese) were excluded.

Using a purposive key-informant sampling approach, survey participants were identified through existing local partners with whom the International Vaccine Institute (IVI) had prior or ongoing collaborations. The online survey link was then disseminated to these identified participants.

### Data management and analysis

Upon survey closure, data were imported into STATA/MP 17.0 for cleaning and analysis. During data cleaning, the records from the participants who had not completed the survey at the time of closure were excluded. Given the descriptive nature of the study, results were summarized primarily using frequencies and percentages and presented in tables and figures to clearly illustrate stakeholders’ perspectives on the questions addressed.

### Ethics statement

The current study received the status of the exempt Institutional Review Board (IRB) review category from the IRB of the International Vaccine Institute (IVI-IRB) (IVI IRB # 2023-007).

## Supplementary information

Supplementary materials

## Data Availability

The datasets generated during the current study are not publicly available due to institutional policies but are available from the corresponding author on reasonable request.
