# Supplementary material for: Need, demand, and feasibility for a new iNTS vaccine: stakeholder insights from eight African countries
Source: NPJ Vaccines. 2026 Feb 11;11:64. doi: 10.1038/s41541-026-01391-2 (PMC13004849; doi:10.1038/s41541-026-01391-2)

Supplementary Files

Supplementary Table 1. Awareness of iNTS disease

Awareness			n (%)
Symptoms identification	Correctly identified	Fever	73 (19.1)
		Cough	11 (2.9)
	Incorrectly identified	Sore throat	9 (2.4)
		Exhaustion	31 (8.1)
		Headache	52 (13.6)
		Rash	4 (1.0)
		Diarrhea	63 (16.5)
		Myalgia	27 (7.0)
		Vomiting	63 (16.5)
		Anorexia	40 (10.5)
		Other	9 (2.4)
Risk factors identification	Direct (host) risk factors identified correctly	Malaria	35 (9.2)
		HIV	32 (8.4)
		Malnutrition	42 (11.1)
		Sickle-cell anemia	22 (5.8)
	Indirect (environmental) risk factors identified correctly	Unsafe drinking water	63 (16.6)
		Contaminated foods	62 (16.4)
		Lack of safe hygiene and sanitation practice	62 (16.4)
	Incorrectly identified	Flies	36 (9.5)
		Contact with a patient with iNTS disease	24 (6.3)
		Other	1 (0.3)
Good practice to avoid iNTS disease		No way to prevent iNTS disease	0 (0.0)
		Boil water before use	62 (15.3)
		Avoid contact with iNTS patients	17 (4.2)
		Maintain proper personal hygiene by washing hands frequently	71 (17.5)
		Maintain clean and proper latrines (or other toilet facilities)	69 (17.0)
		Prevent in-house / in-community water contamination	65 (16.0)
		Take preventive medicines	15 (3.7)
		Avoid eating uncooked (raw) food	55 (13.6)
		Get vaccinated	50 (12.3)
		Other	1 (0.2)
Best ways to treat iNTS patients		No treatment is necessary	2 (1.1)
		Antibiotics	70 (40.2)
		Surgery	9 (5.2)

	Drinking a lot of clean water	26 (14.9)
	Injections	11 (6.3)
	Cleaning the house, environment, or body	30 (17.2)
	Herbal medicines	0 (0.0)
	Vaccination	21 (12.1)
	Other	5 (2.9)
Age group at high risk for iNTS disease	Neonatal	18 (9.7)
	Infants	50 (27.0)
	2-4 years	56 (30.3)
	5-15 years	39 (21.1)
	>15 years	20 (10.8)
	None	2 (1.1)

Supplementary Table 2. Perception on the feasibility of vaccine introduction

Vaccination regimen		n (%)
How many doses would be practical	1 dose	22(29.7)
	2 doses	27(36.5)
	3 doses	24(32.4)
	Other	1(1.4)
The vaccine would be introduced	Universally	38(51.4)
	Subset of the population	36(48.6)

Supplementary Table 3. STROBE Statement—Checklist of items that should be included in reports of *cross-sectional studies*

	Item No	Recommendation	Location where the item is reported
**Title and abstract**	1	(*a*) Indicate the study's design with a commonly used term in the title or the abstract	Title page (Page 1)
		(*b*) Provide in the abstract an informative and balanced summary of what was done and what was found	Page 2
**Introduction**			
Background/rationale	2	Explain the scientific background and rationale for the investigation being reported	Pages 3-4
Objectives	3	State specific objectives, including any prespecified hypotheses	Page 4
**Methods**			
Study design	4	Present key elements of study design early in the paper	Page 4
Setting	5	Describe the setting, locations, and relevant dates, including periods of recruitment, exposure, follow-up, and data collection	Page 4

Participants	6	(*a*) Give the eligibility criteria, and the sources and methods of selection of participants	Page 4
Variables	7	Clearly define all outcomes, exposures, predictors, potential confounders, and effect modifiers. Give diagnostic criteria, if applicable	Figure 1 (Page 6)
Data sources/ measurement	8*	For each variable of interest, give sources of data and details of methods of assessment (measurement). Describe comparability of assessment methods if there is more than one group	Page 4
Bias	9	Describe any efforts to address potential sources of bias	Not applicable (NA)
Study size	10	Explain how the study size was arrived at	Page 7
Quantitative variables	11	Explain how quantitative variables were handled in the analyses. If applicable, describe which groupings were chosen and why	NA
Statistical methods	12	(*a*) Describe all statistical methods, including those used to control for confounding	NA
		(*b*) Describe any methods used to examine subgroups and interactions	NA
		(*c*) Explain how missing data were addressed	Page 5 and Figure 1 (Page 6)
		(*d*) If applicable, describe analytical methods taking account of sampling strategy	NA
		(*e*) Describe any sensitivity analyses	NA
**Results**			
Participants	13*	(a) Report numbers of individuals at each stage of study—eg numbers potentially eligible, examined for eligibility, confirmed eligible, included in the study, completing follow-up, and analysed	Page 7
		(b) Give reasons for non-participation at each stage	Not reported as it was challenging to identify the reasons for not-participation according to the survey type. However, the study has 88.1% participation rate (74/84).
		(c) Consider use of a flow diagram	
Descriptive data	14*	(a) Give characteristics of study participants (eg demographic, clinical, social) and information on exposures and potential confounders	Table 1 (Page 7)
		(b) Indicate number of participants with missing data for each variable of interest	There were no instances of missing

			data reported in this study
Outcome data	15*	Report numbers of outcome events or summary measures	Table 2 & 3 / Figures 2-7
Main results	16	(*a*) Give unadjusted estimates and, if applicable, confounder-adjusted estimates and their precision (eg, 95% confidence interval). Make clear which confounders were adjusted for and why they were included	Tables 2 & 3
		(*b*) Report category boundaries when continuous variables were categorized	NA
		(*c*) If relevant, consider translating estimates of relative risk into absolute risk for a meaningful time period	NA
Other analyses	17	Report other analyses done—eg analyses of subgroups and interactions, and sensitivity analyses	NA
**Discussion**			
Key results	18	Summarise key results with reference to study objectives	Pages 15-16
Limitations	19	Discuss limitations of the study, taking into account sources of potential bias or imprecision. Discuss both direction and magnitude of any potential bias	Page 17
Interpretation	20	Give a cautious overall interpretation of results considering objectives, limitations, multiplicity of analyses, results from similar studies, and other relevant evidence	Pages 15-16
Generalisability	21	Discuss the generalisability (external validity) of the study results	Page 17
**Other information**			
Funding	22	Give the source of funding and the role of the funders for the present study and, if applicable, for the original study on which the present article is based	Page 19

Supplementary Figure 1. Expected iNTS vaccination coverage rates by strategy

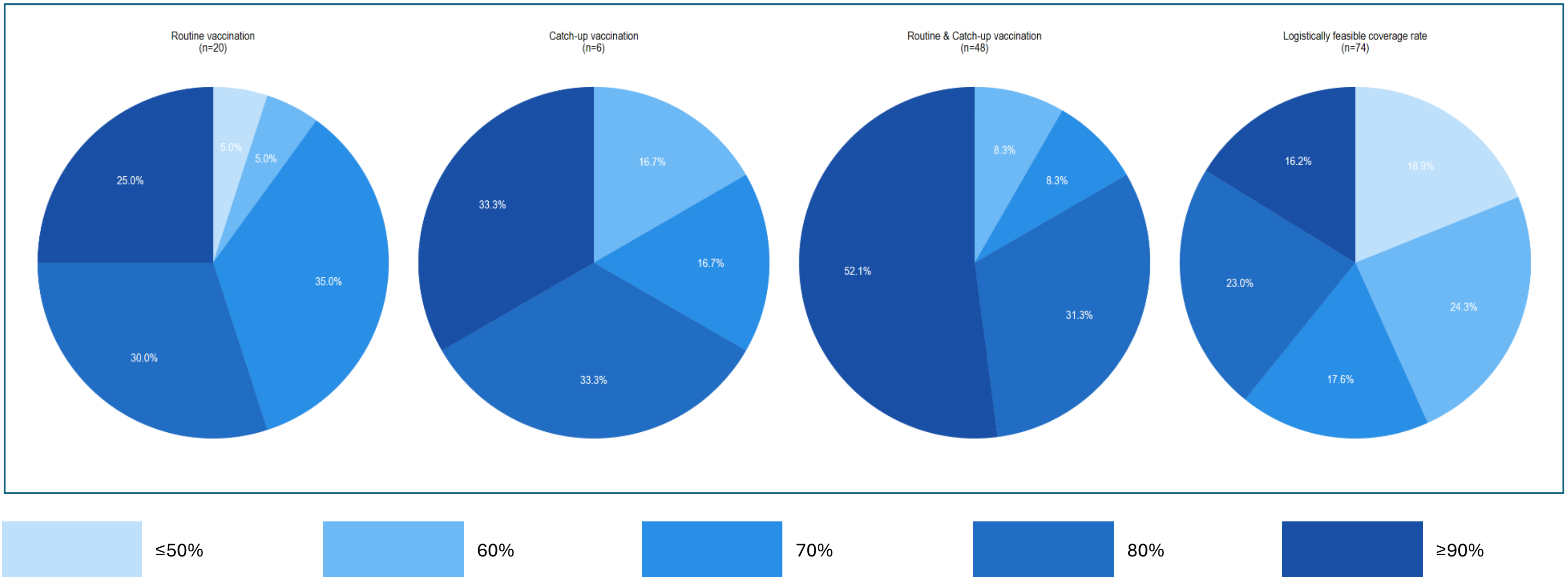

Supplement: Supplementary file 1 — Supplementary materials [file 41541_2026_1391_MOESM1_ESM.pdf]
